# Multiplex PCR assay for the identification of eight *Anopheles* species belonging to the Hyrcanus, Barbirostris and Lindesayi groups

**DOI:** 10.1186/s12936-021-03808-w

**Published:** 2021-06-28

**Authors:** Woo Jun Bang, Heung Chul Kim, Jihun Ryu, Hyeon Seung Lee, So Youn Lee, Myung Soon Kim, Sung Tae Chong, Terry A. Klein, Kwang Shik Choi

**Affiliations:** 1 School of Life Sciences, BK21 FOUR KNU Creative BioResearch Groups, Kyungpook National University, Daegu, 41566 Republic of Korea; 2 Research Institute for Dokdo and Ulleungdo Island, Kyungpook National University, Daegu, 41566 Republic of Korea; 3 Force Health Protection and Preventive Medicine, Medical Department Activity-Korea/65th Medical Brigade, Unit 15281, APO AP, 96271-5281 USA; 4 Research Institute for Phylogenomics and Evolution, Kyungpook National University, Daegu, 41566 Republic of Korea

**Keywords:** *Anopheles*, Multiplex PCR assay, Malaria, Korea

## Abstract

**Background:**

Genus *Anopheles* mosquitoes are the primary vectors of human malaria, which is a serious threat to public health worldwide. To reduce the spread of malaria and identify the malaria infection rates in mosquitoes, accurate species identification is needed. Malaria re-emerged in 1993 in the Republic of Korea (ROK), with numbers peaking in 2004 before decreasing to current levels. Eight *Anopheles* species (*Anopheles sinensis*, *Anopheles pullus*, *Anopheles belenrae*, *Anopheles lesteri*, *Anopheles kleini*, *Anopheles sineroides*, *Anopheles koreicus, Anopheles lindesayi*) are distributed throughout Korea. Members of the *Anopheles* Hyrcanus group currently cannot be identified morphologically. The other species of *Anopheles* can be identified morphologically, except when specimens are damaged in traps. The purpose of this study was to develop a rapid and accurate method for simultaneous molecular identification of the eight *Anopheles* species present in the ROK.

**Methods:**

*Anopheles* spp. used in this study were collected near/in the demilitarized zone in ROK, where most malaria cases are reported. DNA from 165 of the *Anopheles* specimens was used to develop a multiplex PCR assay. The internal transcribed spacer 2 (ITS2) region of each species was sequenced and analysed for molecular identification.

**Results:**

DNA from a total of 165 *Anopheles* specimens was identified to species using a multiplex diagnostic system. These included: 20 *An. sinensis*, 21 *An. koreicus*, 17 *An. lindesayi*, 25 *An. kleini*, 11 *An. lesteri*, 22 *An. sineroides*, 23 *An. belenrae*, and 26 *An. pullus*. Each species was clearly distinguished by electrophoresis as follows: 1,112 bp for *An. sinensis*; 925 bp for *An. koreicus*; 650 bp for *An. lindesayi*; 527 bp for *An. kleini*; 436 bp for *An. lesteri*; 315 bp for *An. sineroides*; 260 bp for *An. belenrae*; and, 157 bp for *An. pullus*.

**Conclusion:**

A multiplex PCR assay was developed to identify *Anopheles* spp. distributed in ROK. This method can be used to accurately identify *Anopheles* species that are difficult to identify morphologically to determine species distributions and malaria infection rates.

## Background

Malaria has a major impact on global public health with more than 200 million people infected and about 4,00,000 deaths annually [1]. Most malaria is reported in Africa (93%), with the remainder reported in Southeast Asia, the Mediterranean, and South America (7%) [2]. Climate change and the expansion of cross-border trading may have contributed to recent increases in malaria risks worldwide [3, 4].

Members of the genus *Anopheles* are vectors of *Plasmodium* spp., the causative agent of malaria. *Plasmodium* spp. that are considered human pathogens include: *Plasmodium falciparum*, *Plasmodium vivax*, *Plasmodium ovale*, *Plasmodium malariae*, and *Plasmodium knowlesi*, the latter previously considered a monkey malaria [5]. In the Republic of Korea (ROK), *P. vivax*, *P. falciparum* and *P. malariae* were eradicated in 1979 by the National Malaria Eradication Service (NMES) of the Korean Government [6], and the World Health Organization (WHO) declared the country malaria free [7]. However, malaria reappeared in 1993 near the demilitarized zone (DMZ) in northern Gyeonggi Province [8]. Except for imported malaria cases, only *P. vivax* is present in ROK and, following its peak of > 4000 cases in 2010, continues to be responsible for 300–500 cases annually [9–11].

In ROK there are eight *Anopheles* species (*Anopheles sinensis*, *Anopheles lesteri*, *Anopheles pullus*, *Anopheles kleini*, *Anopheles sineroides*, and *Anopheles belenrae* belonging to the Hyrcanus group; *Anopheles koreicus* belonging to the Barbirostris group; and, *Anopheles lindesayi* belonging to the Lindesayi group) [12–15]. Recently, two species, *An. lesteri* and *An. kleini*, were proposed to be the primary vectors of malaria in ROK, while *An*. *sinensis* is considered a poor vector. *Anopheles lesteri* showed a large number of *P. vivax* sporozoites (up to 2105) in the salivary glands when compared to *An. sinensis* (0–14) in a single microscope field (750 × 560 μM). Also, *An. kleini* had higher oocyst rates of *P. vivax* (8.8%) in the midgut than *An. sinensis* (4.2%) [15–18]. In another study, while *An. kleini* and *An. sinensis* demonstrated similar numbers of oocysts, *An. kleini* demonstrated + 1 (1–10 sporozoites) to + 4 (> 1000 sporozoites) salivary gland infections, while *An. sinensis* only had + 1 salivary glands [19]. Recent evidence indicates that *An. pullus* and *An. belenrae* are poor to moderate vectors of malaria in ROK (Ubalee, R., pers. comm.). While *An. sineroides* has been implicated as a malaria vector, its status is unknown. Although there are no records of malaria infections in *An. koreicus*, several members of the Barbirostris group are primary vectors of malaria in Southeast Asia [20, 21]. While *An. lindesayi* has not been found positive for malaria in ROK, it has been implicated as a vector of *P. malariae* in Southeast Asia [22]. Accurate identification of *Anopheles* species to determine their distribution and malaria infection rates in order to develop vector control measures is needed in ROK.

Accurate species identification and subsequent monitoring of *Anopheles* spp. is necessary to identify their geographic distributions, larval habitats and population dynamics to manage or conduct epidemiological investigations that identify the most likely sites where infections occurred. Although scales on wings (wing patterns) and spots on legs are used as the primary key characters for species identification, it is extremely difficult if the characters are lost during collections [12, 23]. In addition, *An. sinensis*, *An. lesteri*, *An. kleini*, *An. belenrae*, and *An. pullus* are morphologically very similar and species cannot be identified using current morphological characters [13, 24–26]. Although a multiplex PCR assay to identify six species of the Hyrcanus group was developed [27], molecular diagnostics for all eight *Anopheles* species in ROK had not yet been developed. In this study, a new multiplex PCR assay was developed to identify all *Anopheles* species simultaneously that are present in ROK.

## Methods

### Sample collection

**Fig. 1:**
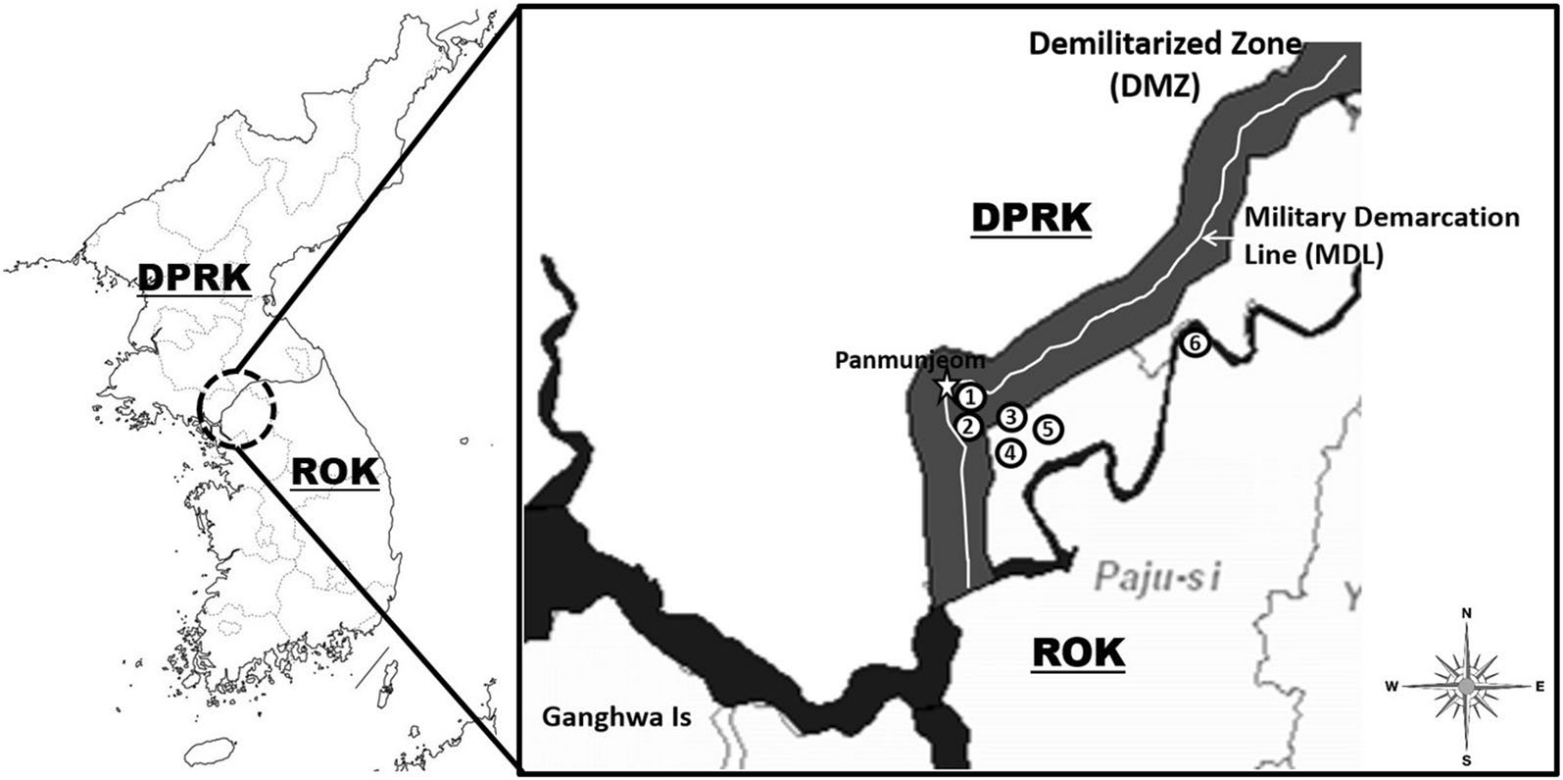
Map of the northern part of Gyeonggi province denoting *Anopheles* collection sites at (**1**) NNSC (Neutral Nations Supervisory Commission camp adjacent to the Panmunjeom); (**2**) Daeseongdong (village of approximately 200 residents inside the DMZ), (**3**) South Gate (South gate entrance to the DMZ) (**4**) Camp Bonifas (US Army installation), (**5**) Warrior Base (US Army training area approximately 3 km from the south gate of DMZ), and (**6**) Dagmar Noth (US Army training area) denoting the six *Anopheles* collection sites in/near the demilitarized zone (DMZ)

Eight species of *Anopheles* mosquitoes used in the study were collected at six sites in/near the DMZ where most malaria infections are contracted: 1) Neutral Nations Supervisory Commission (NNSC) camp adjacent to the Panmunjeom (37°57′17.19″N; 126°40′47.91″E); 2) Daeseongdong (village of approximately 200 residents inside the DMZ (37°56′28.31″N; 126°40′37.38″E)); 3) South Gate (South gate entrance to the DMZ) (37°56′03.53″N; 126°43′15.46″E)); 4) Camp Bonifas (US Army installation (37°55′55.25″N; 126°43′21.73″E)); 5) Warrior Base (US Army training sites approximately 3 km from the south gate of DMZ), (37°55′03.96″N; 126°44′29.74″E)); and, 6) Dagmar North training area (37°58′29.85″N; 126°50′40.88"E). Mosquitoes were collected using Mosquito Magnets® (Woodstream Corp., Lancaster, PA, USA) (Fig. 1). The distance between the two farthest collection points: (2: Daeseongdong and 6: Dagmar North training area) was about 15.2 km. Other points were approximately 3.9 km distant from 3: South gate. Collected mosquitoes were identified morphologically to *Anopheles* spp. [23, 28] and then stored at − 70 °C until used.

### Identification and primer design

Genomic DNA used in this study was extracted using the Chelex protocol [29]. Identification of six species (*An. sinensis*, *An. pullus*, *An. belenrae*, *An. lesteri*, *An. kleini*, *An. sineroides*) was performed using a multiplex PCR assay diagnostic method [27]. The universal primers for the mitochondrial gene cytochrome c oxidase subunit 1 (COI) region (LCO1490: 5′-GGT CAA ATC ATA AAG ATA TTG G-3′/HCO2198: 5′-TAA ACT TCA GGG TGA CCA AAA AAT CA-3′) were used as species identifiers for *An. koreicus* and *An. lindesayi* [30]. Each sample was sequenced by Macrogen (Macrogen Daejeon, Korea) and analysed using BLAST.

Two pairs of universal primers (An-ITS2-U1; forward primer: 5′-ATC GAT GAA GAC CGC AGC TA-3′/reverse primer: 5′-CAA CAC GAC TCC ATG GTA CG-3′; An-ITS2-U2; forward primer: 5′-AAC GGG AGA AGC TCA GCA C-3′/reverse primer: 5′-GAC TTC TTG GTC CGT GTT TCA-3′) were designed between the 5.8 S and 28 S regions of the ribosomal DNA (rDNA) to analyse the entire internal transcribed spacer 2 (ITS2) sequences for the eight *Anopheles* species.

PCR amplification of the whole ITS2 region was conducted as follows. Each individual reaction mixture (25 μl) included: 0.2 mM each dNTP, 0.4 μM each primer, 1X PCR buffer, 1.5 mM MgCl_2_, and 0.5 units *Taq* DNA polymerase (R001AM; TaKaRa, Shiga, Japan) with 1.0 μl genomic DNA extracted from an individual specimen. The PCR cycling conditions were as follows: denaturation at 94 °C for 5 min followed by 35 cycles of denaturation at 94 °C for 30 s, annealing at 55 °C for 40 s, and extension at 72 °C for 60 s; and final extension at 72 °C for 5 min. Each product was visualized on 1.5% (wt/vol) agarose gels stained with ethidium bromide (VWR Life Science, Radnor, PA, USA), and then sequenced in both directions by Macrogen. Sequence data were analysed using Bioedit v7.2.6.1 [31] and deposited in the National Center for Biotechnology Information (NCBI) under the following accession numbers:

*An. sinensis*—MW546412, MW546421; *An. pullus*—MW546424, MW546423; *An. lesteri*—MW546426; *An. sineroides*—MW546417, MW546414; *An. kleini*—MW546419, MW546415; *An. belenrae*—MW546422, MW546418; *An. koreicus*—MW546413, MW546416; *An. lindesayi*—MW546425, MW546420.

### Multiplex PCR assay for eight *Anopheles* species

**Fig. 2:**
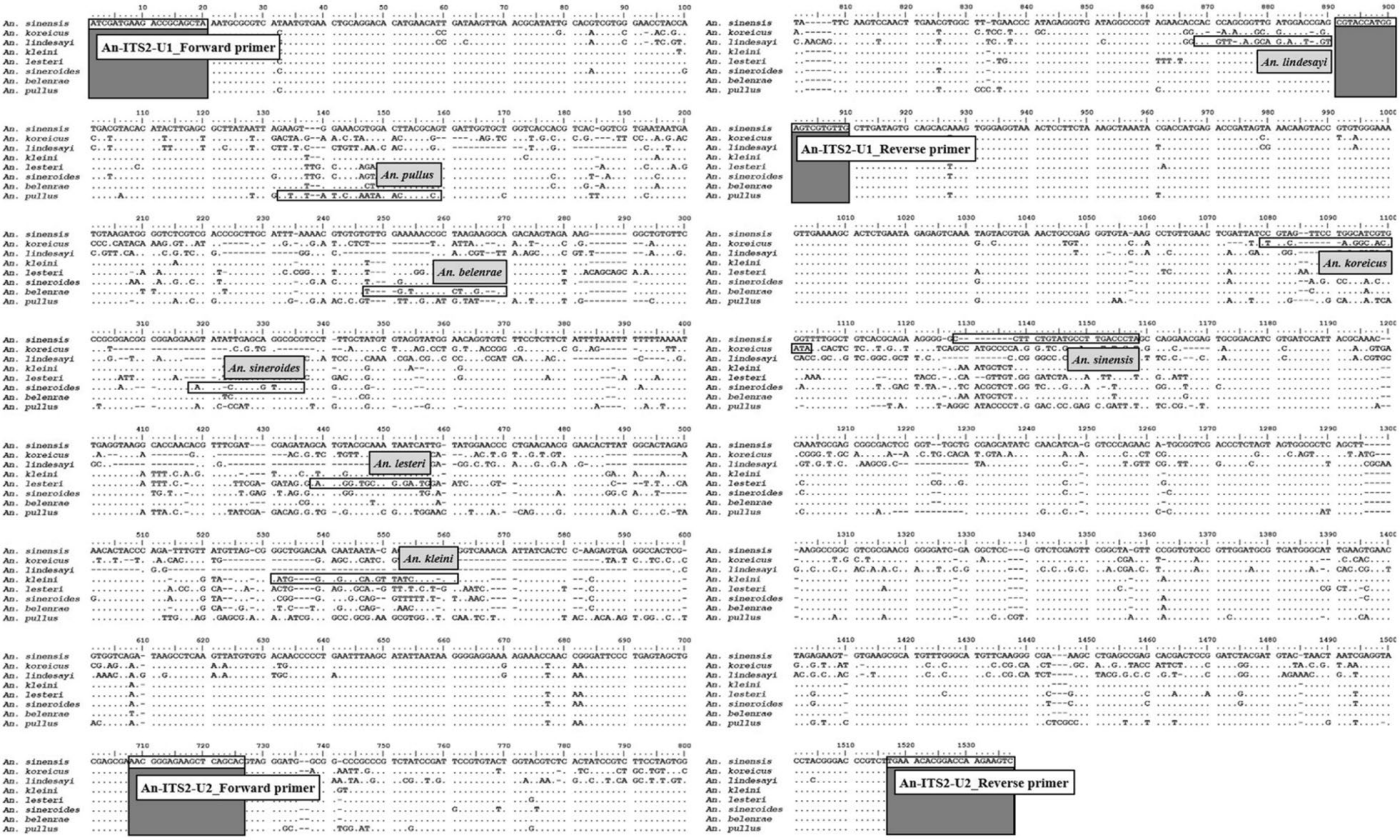
The whole aligned sequences showing positions for two pairs of the universal primers (An-ITS2-U1 and An-ITS2-U2) and the specific reverse primers between 5.8 S and 28 S ribosomal DNA

Universal forward and species-specific reverse primers were designed for the eight species of *Anopheles* present in ROK. Reverse primers for the three species (*An. sinensis*, *An. koreicus*, *An. lindesayi*) were designed using the 28 S rDNA region, while primers for the remaining species were designed using the ITS2 region (Table 1). The multiplex PCR assay was conducted in a 25-μl reaction mixture containing 0.4 μM each primer, 1X PCR buffer, 0.2 mM each dNTP, 0.5 units *Taq* Hotstart DNA polymerase (R007A, TaKaRa,), and 1.0 μl genomic DNA from an individual specimen. PCR amplification was performed under conditions of denaturation at 94 °C for 5 min; 35 cycles of denaturation at 94 °C for 30 s, annealing at 55 °C for 30 s, and extension at 72 °C for 2 min; and final extension at 72 °C for 5 min. The PCR products were visualized on ethidium-bromide–stained 2.0% (wt/vol) agarose gels (VWR Life Science). The whole aligned sequences showing positions for the universal primers and the specific reverse primers are described in Fig. 2.

**Table 1:** PCR primers for the eight *Anopheles* species

Species	Universal forward primer (5′→3′)	Reverse primer (5′→3′)	Product length (bp)
	ATC GAT GAA GAC CGC AGC TA		
*Anopheles sinensis*		TAG GGT CAA GGC ATA CAG AAG G	1112
*Anopheles koreicus*		TAT CGT GGC CCT CGA CAG	925
*Anopheles lindesayi*		ACC ATC TAC TGC CTG AAC GTG	650
*Anopheles kleini*		TTT GTT GAT AAC TTG TAT CGT CCA TC	527
*Anopheles lesteri*		CAG TCT CTT GCA GCC CAT TC	436
*Anopheles sineroides*		CGC GCA CGC TCA GAT ATT	315
*Anopheles belenrae*		TGT CCT AGG CGG TTA TCA ACA	260
*Anopheles pullus*		CGG CGT AGT TTA TTG TGT ATA ACA TC	157

## Results

### Molecular species diagnosis

**Fig. 3:**
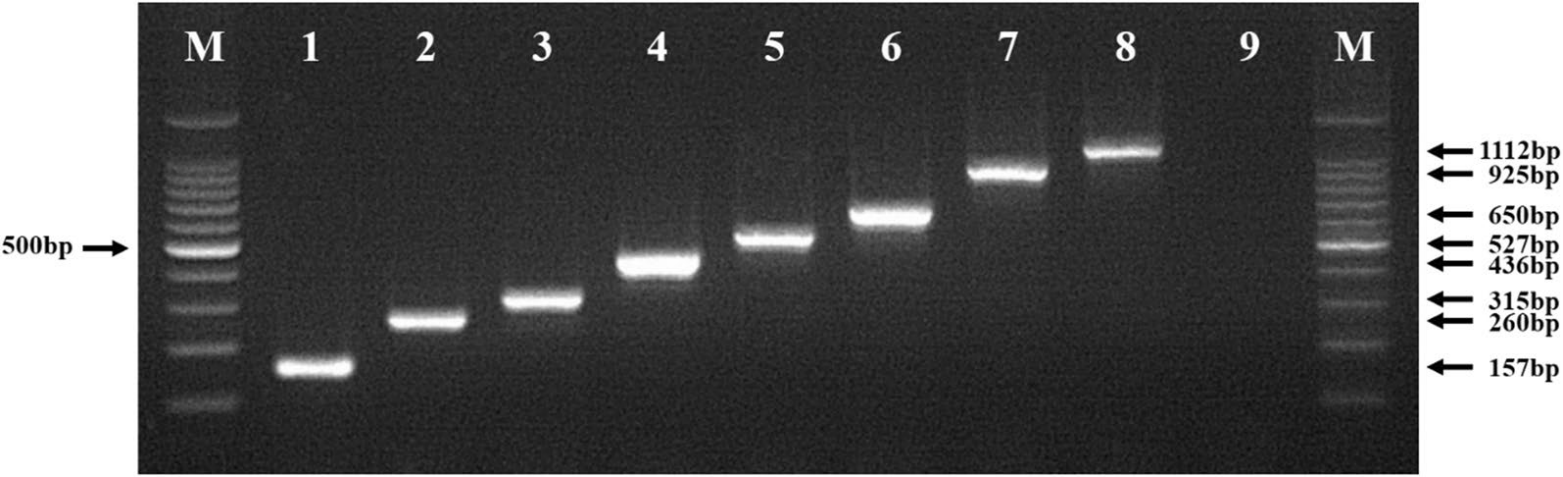
Representative results of agarose gel electrophoretic separation of multiplex PCR products. M: 100 bp molecular marker; lane 1, *An. pullus*; lane 2, *An*. *belenrae*; lane 3, *An*. *sineroides*; lane 4, *An*. *lesteri*; lane 5, *An*. *kleini*; lane 6, *An*. *lindesayi*; lane 7, *An*. *koreicus*; lane 8, *An*. *sinensis*; lane 9, negative control

A total of 165 DNA samples extracted from individual *Anopheles* species were used: *An. sinensis* (20), *An. koreicus* (21), *An. lindesayi* (17), *An. kleini* (25), *An. lesteri* (11), *An. sineroides* (22), *An. belenrae* (23), and *An. pullus* (26). A gel showing the products of multiplex PCR assay separated by agarose gel electrophoresis for the eight species is shown in Fig. 3 (1112 bp for *An. sinensis*; 925 bp for *An. koreicus*; 650 bp for *An. lindesayi*; 527 bp for *An. kleini*; 436 bp for *An. lesteri*,, 315 bp for *An. sineroides*; 260 bp for *An. belenrae;* 157 bp for *An. pullus*). This method allowed identification of all eight *Anopheles* spp., including *An. koreicus* and *An. lindesayi*, and is comparable to the current molecular diagnosis method applied to identify six *Anopheles* species belonging to members of the *Anopheles* Hyrcanus group present in ROK [27]. All samples used in this study were identified using the multiplex assay. The results of species identification for *An. koreicus* and *An. lindesayi*, which were not included in the previous method [27] using this molecular assay, were also consistent with morphological identification results.

### Accurate species identification for the vector control

In Africa and Southeast Asia where malaria is widespread, multiplex PCR assays have been developed and used to identify species accurately and to investigate malaria vector distributions and infection rates [32–40]. In addition, the ITS2-based multiplex PCR assay was used to detect two unknown species (after named as *An. belenrae* and *An. kleini* by Rueda [13]) in ROK [26]. Accurate species identification, using both morphological and molecular methods is important to confirm species identification and monitoring vector populations [41]. Several studies have described accurate species identification as a part of vector surveillance programmes. In India, *Anopheles minimus*, a primary malaria vector, was morphologically misidentified as *Anopheles fluviatilis*, while each species was identified correctly using PCR of the ITS2 regions [42]. In South Africa, *Anopheles vaneedeni* also was reported as a new malaria vector during a malaria surveillance programme using the ITS2 region for specific identification [43]. Molecular diagnostic methods have been used to monitor invasive species, e.g., *Aedes albopictus* and *Aedes aegypti*, to verify morphological identification of specimens, as well as screening for potential new invasive species in Europe [44]. These studies support the importance of accurate species identification for monitoring vector populations and distributions, as well as supporting pathogen surveillance programmes.

### Application of new diagnostic method

The eight *Anopheles* species present in ROK included in three groups (Hyrcanus Barbirostris, Lindesayi) can be identified based on a new multiplex molecular-based method. Morphological identification of these species is challenging, particularly in cases when legs or wing scales used as the primary identification characters are missing or damaged during collections. The method described here enables simple and accurate identification requiring only PCR of individual specimens followed by electrophoresis. It would also be useful to acquire geographic, habitat and population distributions of *An. koreicus* and *An. lindesayi* that are less frequently collected than the other species. Since the re-emergence of vivax malaria in ROK in 1993, most malaria cases have been attributed to exposure near the DMZ. Although the reason for the concentrated outbreak of malaria in/near the DMZ is uncertain, one of the primary vectors, *An. kleini*, is more prevalent near the DMZ than south of Seoul [45]. Additionally, there are reports of higher numbers of malaria cases in the Democratic People’s Republic of Korea (DPRK, North Korea) that provide a source of malaria-infected blood meals for mosquitoes that subsequently migrate south across the DMZ [46–49]. Identification of species distributions and malaria infection rates would assist in understanding the malaria distribution pattern in ROK, in addition to developing vector and malaria mitigation strategies. Recently, two species (*An. lesteri* and *An. kleini*) showed higher infection rates in artificial experiments than *An. sinensis* that was previously considered to be the primary vivax malaria vector in ROK [18, 19]. In China, *An. sinensis* and *An. lesteri* were considered the primary malaria vectors [50]. However, *An. lesteri* demonstrated more anthropophilic behaviour and 20 times higher sporozoite rates (0.58%) than *An. sinensis* (0.02%) [51, 52]. In addition, the annual distribution of *P. vivax* cases varies with environment factors that impact on mosquito population densities, which may be further impacted by climate change [53, 54]. Thus, continuous monitoring of malaria vectors is needed. The new multiplex ITS2-28S rDNA-based method eliminates the requirement for multiple PCR analyses and is useful for monitoring *Anopheles* spp. distributions and population densities in ROK.

## Conclusion

In this study, a new molecular diagnostic method was developed for the identification of eight *Anopheles* spp. present in ROK. This multiplex PCR assay is a simple and accurate method to identify *Anopheles* spp. and can be used as a surveillance tool for monitoring malaria vector population distributions in ROK.

## Data Availability

All data generated during this study are included in the article. Sequences used in this study are deposited in NCBI database (https://www.ncbi.nlm.nih.gov/genbank/) as follow accession numbers: MW546412, MW546421 MW546424 MW546423 MW546426 MW546417 MW546414 MW546419 MW546415 MW546422 MW546418 MW546413 MW546416 MW546425, MW546420.

## References

[1] WHO. Vector-borne diseases. Geneva, World Health Organization. 2020. https://www.who.int/news-room/fact-sheets/detail/vector-borne-diseases. Accessed 17 January 2021.

[2] WHO. World Malaria Report 2019. Geneva: World Health Organization; 2019.

[3] Caminade C, Kovats S, Rocklov J, Tompkins AM, Morse AP, Colón-González FJ (2014). Impact of climate change on global malaria distribution. Proc Natl Acad Sci USA.

[4] Caminade C, Mclnture KM, Jones AE (2019). Impact of recent and future climate change on vector-borne diseases. Ann NY Acad Sci.

[5] Im JH, Kwon HY, Baek J, Park SW, Durey A, Lee KH (2017). Severe *Plasmodium vivax* infection in Korea. Malar J.

[6] Im JH, Kim T-S, Chung M-H, Baek JH, Kwon HY, Lee J-S (2020). Current status and a perspective of mosquito-borne diseases in the Republic of Korea. Vector-Borne Zoonotic Dis.

[7] WHO. Synopsis of the world malaria situation, 1979. Weekly Epidemiological Record. 1981;56: 145–9.

[8] Chai IH, Lim GI, Yoon SN, Oh WI, Kim SJ, Chai JY (1994). Occurrence of tertian malaria in a male patient who has never been abroad. Korean J Parasitol.

[9] Park JW, Klein TA, Lee HC, Pacha LA, Ryu SH, Yeom JS (2003). Vivax malaria: a continuing health threat to the Republic of Korea. Am J Trop Med Hyg.

[10] Kim HC, Pacha LA, Lee WJ, Lee JK, Gaydos JC, Sames WJ (2009). Malaria in the Republic of Korea, 1993–2007. Variables related to re-emergence and persistence of Plasmodium vivax among Korean populations and U.S. forces in Korea. Mil Med.

[11] KDCA. Infectious Disease Portal. Korea Disease Control and prevention Agency, 2021. http://www.kdca.go.kr/npt/biz/npp/nppMain.do. Accessed 17 January 2021.

[12] Tanaka K, Mizusawa K, Saugstad ES (1979). A revision of the adult and larval mosquitoes of Japan (including the Ryukyu Archipelago and the Ogasawara islands) and Korea (Diptera: Culicidae). Contrib Am Entomol Inst.

[13] Rueda LM (2005). Two new species of *Anopheles* (*Anopheles*) hyrcanus group (Diptera: Culicidae) from the Republic of South Korea. Zootaxa.

[14] Foley DH, Klein TA, Chul KIMH, Sames WJ, Wilkerson RC, Rueda LM (2009). Geographic distribution and ecology of potential malaria vectors in the Republic of Korea. J Med Entomol.

[15] Il RH (2005). Studies on *Anopheles sinensis*, the vector species of vivax malaria in Korea. Korean J Parasitol.

[16] Lee WJ, Klein TA, Kim HC, Choi YM, Yoon SH, Chang KS (2007). *Anopheles kleini*, *Anopheles pullus*, and *Anopheles sinensis*: potential vectors of *Plasmodium vivax* in the republic of Korea. J Med Entomol.

[17] Joshi D, Choochote W, Park MH, Kim JY, Kim TS, Suwonkerd W (2009). The susceptibility of *Anopheles lesteri* to infection with Korean strain of *Plasmodium vivax*. Malar J.

[18] Joshi D, Kim JY, Choochote W, Park MH, Min GS (2011). Preliminary vivax malaria vector competence for three members of the *Anopheles* hyrcanus group in the Republic of Korea. J Am Mosq Control Assoc.

[19] Ubalee R, Kim HC, Schuster AL, McCardle PW, Phasomkusolsil S, Takhampunya R, Davidson SA, Lee WJ, Klein TA (2016). Vector competence of *Anopheles kleini* and *Anopheles sinensis* (Diptera: Culicidae) from the Republic of Korea to vivax malaria-infected blood from patients from Thailand. J Med Entomol.

[20] Sinka ME, Bangs MJ, Manguin S, Chareonviriyaphap T, Patil AP, Temperley WH (2011). The dominant *Anopheles* vectors of human malaria in the Asia-Pacific region: occurrence data, distribution maps and bionomic précis. Parasit Vectors.

[21] Amerasinghe PH, Amerasinghe FP, Konradsen F, Fonseka KT, Wirtz RA (1999). Malaria vectors in a traditional dry zone village in Sri Lanka. Am J Trop Med Hyg.

[22] Collins WE, Jeffery GM (2007). *Plasmodium malariae*: parasite and disease. Clin Microbiol Rev.

[23] Ree HI (2003). Taxonomic review and revised keys of the Korean mosquitoes (Diptera: Culicidae). Entomol Res.

[24] Kanda T, Oguma Y (1976). Morphological variations of *Anopheles sinensis* Wiedemann, 1828 and *A. lesteri* Baisas and Hu, 1936 and frequency of clasper movements of the males of several *Anopheles* species during induced copulation. Jap J Sanit Zool.

[25] Ree HI, Yong TS, Hwang UW (2005). Identification of four species of the *Anopheles* hyrcanus complex (Diptera: Culicidae) found in Korea using species–specific primers for polymerase chain reaction assay. Med Entomol Zool.

[26] Li C, Lee JS, Groebner JL, Kim HC, Klein TA, O’guinn ML (2005). A newly recognized species in the *Anopheles* hyrcanus group and molecular identification of related species from the Republic of South Korea (Diptera: Culicidae). Zootaxa.

[27] Joshi D, Park MH, Saeung A, Choochote W, Min GS (2010). Multiplex assay to identify Korean vectors of malaria. Mol Ecol Resour.

[28] Lee KW. A revision of the illustrated taxonomic keys to genera and species of female mosquitoes of Korea (Diptera: Culicidae). Yongsan: U S Army. 2001; p. 40.

[29] Musapa M, Kumwenda T, Mkulama M, Chishimba S, Norris DE, Thuma PE (2013). A simple Chelex protocol for DNA extraction from *Anopheles* spp. J Vis Exp.

[30] Folmer O, Black M, Hoeh W, Lutz R, Vrijenhoek R (1994). DNA primers for amplification of mitochondrial cytochrome c oxidase subunit I from diverse metazoan invertebrates. Mol Mar Biol Biotechnol.

[31] Hall TA (1999). BioEdit: a user-friendly biological sequence alignment editor and analysis program for Windows 95/98/NT. Nucleic Acids Symp Ser.

[32] Scott JA, Brogdon WG, Collins FH (1993). Identification of single specimens of the *Anopheles gambiae* complex by the polymerase chain reaction. Am J Trop Med Hyg.

[33] Beebe NW, Saul A (1995). Discrimination of all members of the Anopheles punctulatus complex by polymerase chain reaction–restriction fragment length polymorphism analysis. Am J Trop Med Hyg.

[34] Fanello C, Santolamazza F, della Torre A. (2002). Simultaneous identification of species and molecular forms of the *Anopheles gambiae* complex by PCR-RFLP. Med Vet Entomol.

[35] Mohanty A, Kar P, Mishra K, Singh DV, Mohapatra N, Kar SK (2007). Multiplex PCR assay for the detection of *Anopheles fluviatilis* species complex, human host preference, and *Plasmodium falciparum* sporozoite presence, using a unique mosquito processing method. Am J Trop Med Hyg.

[36] Brosseau L, Udom C, Sukkanon C, Chareonviriyaphap T, Bangs MJ, Saeung A (2019). A multiplex PCR assay for the identification of five species of the *Anopheles barbirostris* complex in Thailand. Parasit Vectors.

[37] Koekemoer LL, Kamau L, Hunt RH, Coetzee M (2002). A cocktail polymerase chain reaction assay to identify members of the *Anopheles funestus* (Diptera: Culicidae) group. Am J Trop Med Hyg.

[38] Phuc HK, Ball AJ, Son L, Hanh NV, Tu ND, Lien NG (2003). Multiplex PCR assay for malaria vector *Anopheles minimus* and four related species in the Myzomyia series from Southeast Asia. Med Vet Entomol.

[39] Walton C, Handley JM, Kuvangkadilok C, Collins FH, Harbach RE, Baimai V (1999). Identification of five species of the *Anopheles dirus* complex from Thailand, using allele-specific polymerase chain reaction. Med Vet Entomol.

[40] Garros C, Koekemoer LL, Coetzee M, Coosemans M, Manguin S (2004). A single multiplex assay to identify major malaria vectors within the african *Anopheles funestus* and the oriental *An. minimus* groups. Am J Trop Med Hyg.

[41] Erlank E, Koekemoer LL, Coetzee M (2018). The importance of morphological identification of African anopheline mosquitoes (Diptera: Culicidae) for malaria control programmes. Malar J.

[42] Singh OP, Nanda N, Dev V, Bali P, Sohail M, Mehrunnisa A (2010). Molecular evidence of misidentification of *Anopheles minimus* as *Anopheles fluviatilis* in Assam (India). Acta Trop.

[43] Burke A, Dandalo L, Munhenga G, Dahan-Moss Y, Mbokazi F, Ngxongo S (2017). A new malaria vector mosquito in South Africa. Sci Rep.

[44] Ibáñez-Justicia A, Smitz N, Den Hartog W, van de Vossenberg B, De Wolf K, Deblauwe I (2020). Detection of exotic mosquito species (Diptera: Culicidae) at international airports in Europe. Int J Environ Res Public Health.

[45] Kim HC, Klein TA, Lee WJ, Collier BW, Chong ST, Sames WJ (2007). Mosquito species distribution and larval breeding habitats with taxonomic identification of Anopheline mosquitoes in Korea. Entomol Res.

[46] Chai JY (2020). History and current status of malaria in Korea. Infect Chemother.

[47] Yoo DH, Shin EH, Park MY, Kim HC, Lee DK, Lee HH (2013). Short Report: Mosquito species composition and *Plasmodium vivax* infection rates for Korean army bases near the demilitarized zone in the Republic of Korea, 2011. Am J Trop Med Hyg.

[48] Chang KS, Yoo DH, Ju YR, Lee WG, Roh JY, Kim HC (2016). Distribution of malaria vectors and incidence of vivax malaria at Korean army installations near the demilitarized zone. Republic of Korea Malar J.

[49] Ree HI (2000). Unstable vivax malaria in Korea. Korean J Parasitol.

[50] Zhang S, Guo S, Feng X, Afelt A, Frutos R, Zhou S (2017). *Anopheles* vectors in mainland China while approaching malaria elimination. Trends Parasitol.

[51] Zhu G, Xia H, Zhou H, Li J, Lu F, Liu Y (2013). Susceptibility of *Anopheles sinensis* to *Plasmodium vivax* in malarial outbreak areas of central China. Parasit Vectors.

[52] Liu C (1990). [Comparative studies on the role of *Anopheles anthropophagus* and *Anopheles sinensis* in malaria transmission in China](in Chinese). Zhoughua Liu Xing Bing Xue Za Zhi.

[53] Park JW (2011). Changing transmission pattern of *Plasmodium vivax* malaria in the Republic of Korea: relationship with climate change. Environ Health Toxicol.

[54] Mihailović DT, Petrić D, Petrović T, Hrnjaković-Cvjetković I, Djurdjevic V, Nikolić-Đorić E (2020). Assessment of climate change impact on the malaria vector *Anopheles hyrcanus*, West Nile disease, and incidence of melanoma in the Vojvodina Province (Serbia) using data from a regional climate model. PLoS ONE.

